# Combination of Gemcitabine with Cell-Penetrating Peptides: A Pharmacokinetic Approach Using in Silico Tools

**DOI:** 10.3390/biom9110693

**Published:** 2019-11-04

**Authors:** Abigail Ferreira, Rui Lapa, Nuno Vale

**Affiliations:** 1 Laboratory of Pharmacology, Department of Drug Sciences, Faculty of Pharmacy, University of Porto, Rua de Jorge Viterbo Ferreira, 228, 4050-313 Porto, Portugal; nuno.vale@ff.up.pt; 2 LAQV/REQUIMTE, Laboratory of Applied Chemistry, Department of Chemical Sciences, Faculty of Pharmacy, University of Porto, Rua de Jorge Viterbo Ferreira, 228, 4050-313 Porto, Portugal; ruilapa@ff.up.pt; 3 Institute of Molecular Pathology and Immunology of the University of Porto (IPATIMUP), Rua Júlio Amaral de Carvalho, 45, 4200-135 Porto, Portugal; 4 Instituto de Investigação e Inovação em Saúde (i3S), University of Porto, Rua Alfredo Allen, 208, 4200-135 Porto, Portugal; 5 Department of Molecular Pathology and Immunology, Abel Salazar Biomedical Sciences Institute (ICBAS), University of Porto, Rua de Jorge Viterbo Ferreira, 228, 4050-313 Porto, Portugal

**Keywords:** gemcitabine, cell-penetrating peptides (CPP), in silico, pharmacokinetics, GastroPlus™, z-scale

## Abstract

Gemcitabine is an anticancer drug used to treat a wide range of solid tumors and is a first line treatment for pancreatic cancer. Our group has previously developed novel conjugates of gemcitabine with cell-penetrating peptides (CPP), and here we report some preliminary data regarding the pharmacokinetics of gemcitabine, two gemcitabine-CPP conjugates and respective CPP gathered from GastroPlus™, and analyze these results considering our previous evaluation of gemcitabine release and conjugates’ bioactivity. Additionally, seeking to shed some light on the relation between the penetration ability of CPP and their physicochemical properties, chemical descriptors for the 20 natural amino acids were calculated, a new principal property scale (z-scale) was created and CPP prediction models were developed, establishing quantitative structure-activity relationships (QSAR). The z-scores of the peptides conjugated with gemcitabine are presented and analyzed with the aforementioned data.

## 1. Introduction

**Figure 1:**
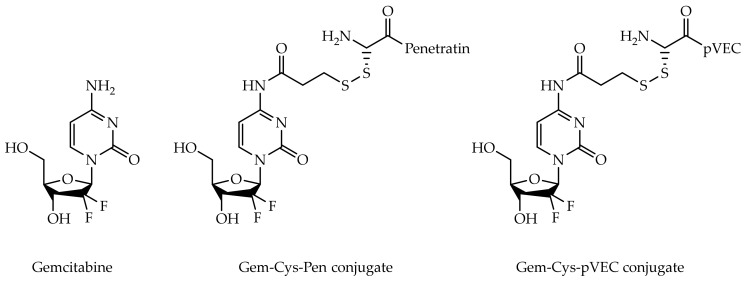
Chemical structures of gemcitabine and gemcitabine-CPP conjugates Gem-Cys-Pen and Gem-Cys-pVEC.

Gemcitabine (2′,2′-difluoro-2′-deoxycytidine, dFdC, Gem, Figure 1) is a drug considered as ‘first-line treatment’ for various types of solid tumors and is clinically used in the treatment of various cancers including pancreatic cancer, non-small cell lung cancer (NSCLC), bladder, ovarian, and breast cancer, as well as some blood cancers, such as non-Hodgkin’s lymphoma [1,2,3]. Like most anticancer drugs, gemcitabine is administered intravenously. Its cellular uptake is primarily facilitated and governed by the human equilibrative nucleoside transporter 1 (hENT1) and human concentrative nucleoside transporter 3 (hCNT3) [4,5]. Inside cells, gemcitabine acts as an antimetabolite, but first needs to be activated by phosphorylation to its triphosphate form (dFdCTP) by deoxycytidine kinase (dCK) and other intracellular kinases. dFdCTP is incorporated into DNA, leading to DNA strand termination after the incorporation of one more nucleotide, and also competes with deoxycytidine triphosphate (dCTP) as an inhibitor of DNA polymerase. The incorporation of this extra nucleotide into DNA appears to be resistant to the normal mechanisms of DNA. Moreover, gemcitabine diphosphate (dFdCdP) is a potent inhibitor of ribonucleotide reductase (RNR), resulting in depletion of deoxyribonucleotides necessary for DNA synthesis, further potentiating the effects of dFdCTP in causing cell death by apoptosis [6,7].

However, there are some factors hindering the full potential of gemcitabine: (a) gemcitabine may rapidly undergo deamination to its inactive uridine metabolite (2’,2’-difluorodeoxyuridine, dFdU), by cytidine deaminase (CDA), which is present at high levels in both human plasma and liver; (b) gemcitabine monophosphate (dFdCMP) is deaminated into dFdUMP by deoxycytidylate deaminase (DCTD); (c) the phosphorylated metabolites of gemcitabine are inactivated via reduction by cellular 5’-nucleotidase (5’-NT). Enzymatic conversion of gemcitabine rapidly clears it from the body [7,8]. Additionally, some tumor cells can develop resistance to gemcitabine related to nucleoside transporter deficiency [5]. As the adverse effects associated with chemotherapeutic agents remain severe, many efforts have been made to maximize therapeutic efficacy and attenuate the nocuous side manifestations. Numerous gemcitabine prodrugs have been developed to alter some of the unfavorable physicochemical properties of the drug and ideally improve its oral bioavailability.

Recently, our group has synthesized two gemcitabine-CPP conjugates (Figure 1), in an effort to both retard or prevent deamination of gemcitabine (masking its aniline moiety) and facilitate its delivery into cancer cells [9], taking advantage of the fact that all CPP are able to efficiently pass through cell membranes while being non-cytotoxic and carrying a wide variety of cargos inside cells [9,10]. CPP Penetratin (Pen, RQIKIWFQNRRMKWKK-NH_2_) and pVEC (LLIILRRRIRKQAHAHSK-NH_2_) were selected for conjugation with gemcitabine [9]. These are two well-known CPP and have both been reported in numerous cancer studies over the last two decades [11,12]. An additional cysteine residue (Cys) was coupled to the N-terminus of both CPP, producing Cys-Pen and Cys-pVEC, to allow the subsequent binding to parent drug. The time-dependent kinetics of gemcitabine release from hydrolysis of these new conjugates was studied in phosphate-buffered saline (PBS) at pH 7.4, 37 °C, and their biological activity was evaluated against three human tumoral cell lines: MKN-28 (human gastric cancer), Caco-2 (heterogeneous human epithelial colorectal adenocarcinoma) and HT-29 (human colon adenocarcinoma). The results were promising, revealing an increase in the anti-proliferative activity of gemcitabine in vitro upon conjugation with the CPP [9].

In this work, we used computational tools to study the pharmacokinetics (PK) of drug gemcitabine, gemcitabine-CPP conjugates and respective CPP, and to establish a possible relation between penetration potency of CPP and their physicochemical properties. The PK data was acquired using GastroPlus™; amino acid properties were calculated in Schrödinger’s Maestro software; principal component analysis (PCA), multivariate analysis (MVA) and partial least square discriminant analysis (PLS-DA) were used to build CPP prediction models in SIMCA by Umetrics. GastroPlus™ is a powerful mechanistically based simulation and modeling software for pharmaceutical research. With Advanced Compartmental Absorption and Transit (ACAT™) and Physiologically Based Pharmacokinetic (PBPK) models, it has features and capabilities to support model-based drug development in all phases of drug discovery, translational research, and clinical development. This software has been used in numerous academic studies and by pharmaceutical companies of excellence, along with the U.S. Food and Drug Administration (FDA), the Centers for Disease Control and Prevention (CDC), the National Institutes of Health (NIH), the National Cancer Institute (NCI) and the China Food and Drug Administration (CFDA).

Peptides and proteins have been the subject of considerable interest in medicine, research, and drug development due to some of their specific properties and a wide variety of applications [13,14]. In particular, CPP have the intrinsic property to efficiently deliver covalently or noncovalently bound therapeutic molecules (nucleic acids, proteins, drugs, imaging agents, etc.) into a variety of cell types and tissues in a nontoxic manner, via receptor-independent mechanisms (primarily endocytosis) [10,15,16]. Besides their ability to be uptaken by cells and act as an excellent therapeutic delivery vehicle, it has been established that CPP are generally relatively short peptides (less than 40 amino acids), have low cytotoxicity, dose-dependent efficiency, and no restriction with respect to the size or type of cargo. Additionally, CPP can enhance the water solubility of drugs [17].

The rational design and prediction of new CPP requires an understanding of the defining properties and similarities of these peptides. For example, almost every CPP sequence involves positively charged amino acids and it has also been shown that secondary structure, specifically helicity, is a key factor governing the interactions of a given CPP with cell membranes, and peptides with an α-helical region can enter cells more efficiently [18].

Theoretical and computational methods are powerful and very often useful tools to predict new CPP sequences, based on previously available experimental data and calculations of several amino acid and peptide properties. Initially, principal components analysis (PCA) and binary classification were explored for pattern recognition models [19,20]. With the determination of physicochemical properties of amino acids and peptides, quantitative structure activity relationship studies (QSAR), partial least squares (PLS) regression and multivariate analysis (MVA) can also be used as tools [21,22,23,24]. Hellberg et al. developed a tridimensional scale (z1–z3) for the 20 natural amino acids to perform quantitative structure-activity relationship (QSAR) of peptides, using 29 physicochemical properties [23]. This method and these scales have since been extended to include more amino acids and descriptive properties in the search for new CPP sequences.

In this project, we followed the same methodology and selected 12 physicochemical properties of the 20 natural amino acids to extract 3 z-scores. This tridimensional z-scale was used to build several CPP prediction models and to discuss the properties of amino acids and peptides that seem to play an important role in the penetration ability of these peptide sequences. Although it is possible to create models that allow for amino acid position-based optimization, the models created here were to predict a binary classification: CPP or non-CPP, using various calculated global peptide descriptors. This has been applied in multiple previous studies regarding peptide modeling with varying successful results [21,25].

## 2. Methods

### 2.1. Amino Acids–Structure, Physicochemical Properties and Creation of a Z-Scale

The structures of the 20 natural amino acids were drawn in Maestro (version 10.4, Schrödinger, LLC, New York, NY, USA). All amino acids were capped using C-terminal amidation and *N*-terminal acetylation to better simulate an amino acid as part of a peptide chain, linked through amide bonds. Maestro’s LigPrep tool was used to simultaneously minimize the structures and generate possible charge states at pH 7.0 (histidine was only included in its charged, deprotonated state).

Physicochemical properties of the amino acids were calculated by Maestro’s QikProp tool. This data was imported into SIMCA (version 13.0 ed, Umetrics AB, Umeå, Sweden) where it was scaled and centered. After principal component analysis (PCA) of the data, 3 principal components were extracted. These scores (designated z1, z2 and z3) constitute a z-scale used to quantitatively describe each amino acid and the peptide sequences. The 12 selected properties for PCA were: number of rotatable bonds (#rotor), molecular weight (mol MW), volume, solvent accessible surface area (SASA), number of hydrogen bond donors (donorHB), number of hydrogen bond acceptors (accptHB), globularity (glob), octanol/water partition coefficient (QPlogPo/w), polar surface area (PSA), net charge (Tot Q), and ratios FISA/SASA (FISA is the hydrophilic component of SASA) and FOSA/SASA (FOSA is the hydrophobic component of SASA).

In general, the relation of the PCs with the physicochemical properties suggests the first PC (z1) is mainly related to properties describing size and shape properties, such as volume, SASA, globularity and molecular weight; the second PC (z2) seems to be more related to the polarity of the amino acids and the descriptors QPlogPo/w, accptHB, donorHB, FISA/SASA and PSA; finally, z3 seems to be predominantly influenced by electronic properties (in this case described by charge).

### 2.2. Peptides

#### 2.2.1. Datasets

Peptide sequences were extracted from the different CellPPD (Designing of Cell Penetrating Peptides) databases, available from http://crdd.osdd.net/raghava/cellppd/dataset.php. This provided a main dataset of experimentally validated cell-penetrating (900 CPP) and both validated and randomly generated non-active peptides (1148 non-CPP) after removal of duplicates.

The lack of experimentally validated non-CPP is a known problem [26,27] and creating balanced datasets, which has been demonstrated to be very crucial in modeling [26,28,29], is therefore a major problem. To try to overcome this issue, the main dataset was reduced to contain only 900 non-CPP, the same number of CPP present. The deleted 248 peptides were selected randomly.

To study the influence of terminal chains, all peptides were truncated to originate 6 other datasets. First, the peptides were divided in half, generating an N-terminal and a C-terminal dataset. In the cases of peptides with an odd number of amino acids, the N-terminal was the longer chain. Then, five residues were taken from each terminal, originating the “first 5AA” and “last 5AA” datasets. Finally, the same process was used, but to create “first 10AA” and “last 10AA” datasets.

#### 2.2.2. Peptide Descriptors

Every peptide is described as a sequence of the z-scores of their amino acids. The mean of the z-scores across the entire sequence was calculated (mean z1, mean z2 and mean z3). The absolute difference between terminals was calculated (|Nt − Ct|), as well as the absolute difference between the first and last 5 or 10 amino acids. Using an extension of the Eisenberg’s equation where the hydrophilicity descriptor of the original equation was replaced with the generated z-scale values (Equation (1)), as established by Maccari et al. [30], the z-scale moment was calculated for each dataset.

Equation (1): Original Eisenberg’s equation; N: number of amino acids in the peptide sequence; n: order number of the specific amino acid examined; H: experimental hydrophilicity of a specific amino acid; δ: angle between two adjacent amino acids, which in the case of an alpha helical structure is defined as 100°.

(1) $$\mu =\sqrt{{\left(\overset{N}{\underset{n=1}{\sum }}H_{n}\cdot \sin\left(\delta n\right)\right)}^{2}+{\left(\overset{N}{\underset{n=1}{\sum }}H_{n}\cdot \cos\left(\delta n\right)\right)}^{2}}$$

Some properties of the peptides were calculated and applied as descriptors to the models. These properties included the peptides’ steric bulk (calculated as the mean number of non-hydrogen atoms in the amino acid side chains), the mean net donating hydrogen bonds (calculated as the accepted hydrogen bonds subtracted from the donated hydrogen bonds), the total charge of the peptide sequences as well as the mean net charge, which takes into consideration the total number of amino acids in each sequence. Additionally, the total number of Arg, His, Lys, Asp and Glu residues, and the total number and ratio of positively and negatively charged amino acids were also considered when building the prediction models.

### 2.3. Prediction Model Generation and Optimization

Using SIMCA and Partial Least Square Discriminant Analysis (PLS-DA), numerous prediction models were generated by varying the included properties and descriptors.

To be able to perform external validation of the built models, a test set composed of peptides not included in the generation of the models is needed. So, 50% of the main dataset peptides were randomly extracted and selected as the test set.

Internal classification predictive value, Q^2^, and fit measurements were calculated and analyzed in the optimization process. Four performance measurements to access the predictability of the different models were calculated based on the number of true positives (TP), true negatives (TN), false positives (FP) and false negatives (FN). These measurements were sensitivity, specificity, accuracy and Matthew’s correlation coefficient (MCC), a quality measurement for binary classifications (Equations (2) to (5)).

Equations (2) to (5): Sensitivity, representing the percentage of correctly predicted positive sequences; specificity, representing the percentage of correctly predicted negative sequences; accuracy, representing the percentage of correctly predicted sequences overall; and Matthew’s correlation coefficient (MCC), a quality measurement for binary classifications.

(2) $$\left(\mathrm{Sensitivity}\right):\;\frac{\mathrm{TP}}{\mathrm{TP}+\mathrm{FN}}\times 100$$

(3) $$\left(\mathrm{Specificity}\right):\;\frac{\mathrm{TN}}{\mathrm{TN}+\mathrm{FP}}\times 100$$

(4) $$\left(\mathrm{Accuracy}\right):\;\frac{\mathrm{TP}+\mathrm{TN}}{\mathrm{TP}+\mathrm{FN}+\mathrm{TN}+\mathrm{FP}}\times 100$$

(5) $$\left(\mathrm{MCC}\right):\;\frac{\left(\mathrm{TP}\times \mathrm{TN}\right)-\left(\mathrm{FP}\times \mathrm{FN}\right)}{\sqrt{\left(\mathrm{TP}+\mathrm{FP}\right)\times \left(\mathrm{TP}+\mathrm{FN}\right)\times \left(\mathrm{TN}+\mathrm{FP}\right)\times \left(\mathrm{TN}+\mathrm{FN}\right)}}\times 100$$

### 2.4. Pharmacokinetic Assessment of Gemcitabine, Cpp and Gemcitabine-Cpp Conjugates

The pharmacokinetic study of gemcitabine, CPP and conjugates was performed in GastroPlus™ (version 9.5, Simulations Plus, Inc., Lancaster, California, USA), a mechanistically based simulation and modeling software for pharmaceutical research. GastroPlus™ builds physiologically based pharmacokinetic (PBPK) models and can run simulations based on a drug’s structure and collected data to predict the most important parameters in pharmacokinetics (PK), such as the maximum concentration reached in plasma and liver, time necessary to reach such concentrations, binding to plasma proteins, fraction absorbed and bioavailability. It also draws a graphical representation of plasmatic concentration over time and calculates the area under the curve (AUC). GastroPlus™ not only simulates human PK, but can also be used to study mice, rats, monkeys, beagles, cats, rabbits and minipigs, based on preinstalled human and animal physiological parameters. This software has been used to successfully and accurately predict PK profiles, an important tool in early on drug discovery [31].

All the simulations in the scope of this project were performed to predict the PK for 24 h after intravenous administration of 1250 mg (1 h perfusion), using the Compartmental model of GastroPlus™. The software did not provide an estimated clearance for any of the molecules studied here, thus, gemcitabine’s clearance value of 168 L/h was input into the software, according to this drug’s FDA label and information deposited on DrugBank [32,33,34].

The general workflow of PBPK modeling has been described in publications and tutorials [35,36,37]. The preliminary model in this case was based on the physicochemical data from ADMET Predictor™ module of GastroPlus™, using a standard compartmental PBPK model.

## 3. Results and Discussion

**Figure 2:**
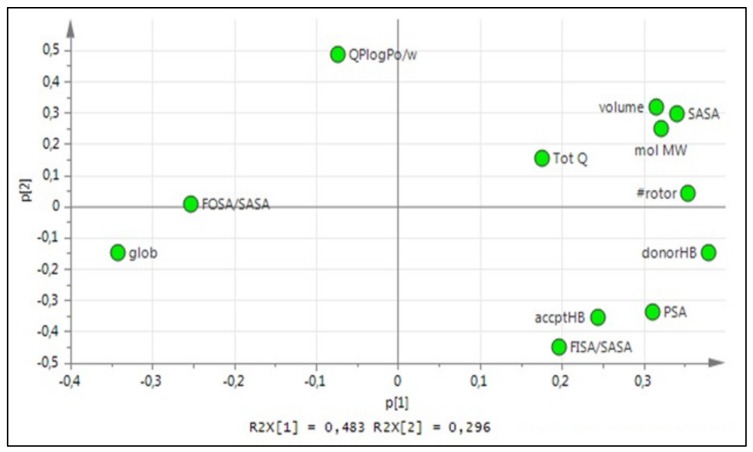
Loading plot explaining PC1 vs. PC2 for the 20 natural amino acids z-scale PCA.

The choice of amino acids and their combination in a peptide sequence when designing new CPP are fundamental. Properties such as size, polarity and charge vary greatly within the 20 natural amino acids, and to better understand how these properties correlate with CPP penetration ability, 12 physicochemical properties were selected and PCA was performed to extract 3 principal components (PCs), forming a tridimensional z-scale, presented in Table 1. The relation of the PCs with the physicochemical properties can be seen in Figure 2.

Every peptide was described as a sequence of the z-scores of their amino acids and peptide descriptors were calculated for every peptide in the main dataset. PCA was performed to extract 3 PCs for each peptide. Several prediction models were generated by varying the included properties and descriptors, and Pen, Cys-Pen, pVEC and Cys-pVEC were predicted as CPP; their z-scores are presented in Table 2. All the models created in this project showed a decent ability to predict CPP, with an average of 79% sensitivity, 80% specificity, 81% accuracy, 61% MCC and 0.406 Q^2^.

These results show Pen and Cys-Pen have z1 scores 2-fold higher than the z1 scores calculated for pVEC and Cys-pVEC. The same difference was observed for the z2 scores. However, regarding the third PC, pVEC and Cys-pVEC z3 scores are higher than the ones calculated for Pen and Cys-Pen. Adding a Cys residue to the original CPP sequences seems to have had a bigger impact on the PC related to size and shape, z1, where it was possible to differentiate the original CPP from the modified Cys-CPP, whereas z2 and z3 scores are very similar for the CPP and Cys-CPP.

As previously mentioned, charged has long been appointed as one of the most important features/characteristics of CPP. In Table 3, the number of charged amino acids and ratio of hydrophilic residues to total number of residues are presented. The difference in the content of positively charged amino acids in Pen and pVEC can explain the higher z3 scores calculated for pVEC (and Cys-pVEC).

With respect to the in vitro results previously observed by our group, there was a significant improvement in the biological activity of gemcitabine upon conjugation of the drug with either CPP, with Gem-Cys-pVEC conjugate showing the best results in MKN-28 and HT-29 cells (Table 4).

In Table 5 are the input data used in GastroPlus^TM^ to simulate plasma concentration. Concentration curves were then compared to that of parental drug (GEMZAR^®^, gemcitabine for injection) and the approximation between values has been achieved.

Despite the promising in vitro bioactivity, favorable pharmacokinetic properties are required for the success of therapies in vivo. According to the simulations carried out in GastroPlus™, conjugates’ bioavailability is ensured and plasma concentration should reach therapeutic levels (Table 6).

**Figure 3:**
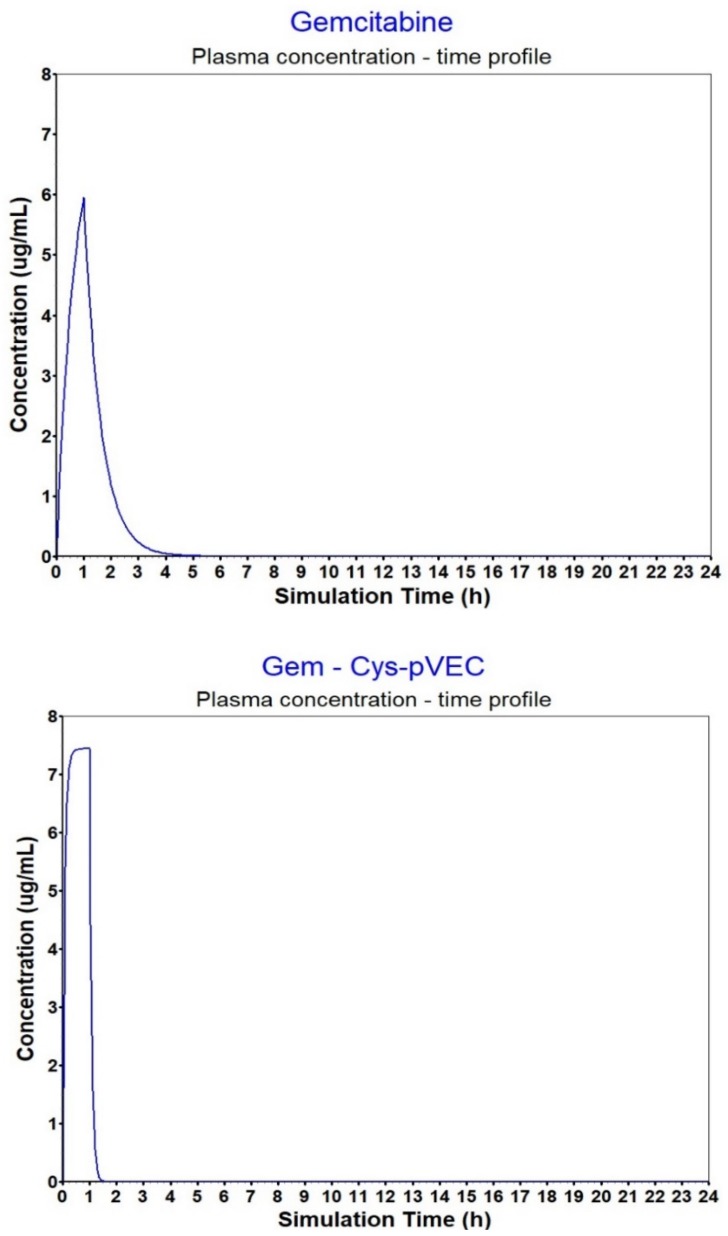
Plasma concentration–time profiles for gemcitabine and the Gem-Cys-pVEC conjugate after IV infusion (1 h).

The calculated AUC for the conjugates was comparable to the AUC calculated for gemcitabine, yet, estimated C_max_ was higher for all peptides and conjugates analyzed compared to gemcitabine alone (Figure 3). Gem- Cys-pVEC conjugate binds less extensively to plasma proteins (>F_up_, 42.89%). Considering this conjugate showed the best bioactivity in MKN-28 and HT-29 cells, and released gemcitabine in PBS faster than Gem-Cys-Pen conjugate (50% over 42 h, versus 9.6 days for Gem-Cys-Pen [8]), we believe Gem-Cys-pVEC conjugate has the best suitable profile for drug delivery. Binding to plasma proteins acts as a protection from quick biotransformation and degradation due to the action of plasma circulating enzymes [40] (such as proteases and CDA). This increases circulation time and can also be advantageous to biodistribution. However, it is important that there is a significant percentage/amount of the drug/compound free in circulation so that it can reach its target and exert its pharmacologic action. Differences in V_c_ of gemcitabine and the conjugates can be explained by their different affinity to bind to plasma proteins.

## 4. Conclusions

The main goal of this study was to combine the in vitro and in silico approaches to highlight the potential clinical applications of CPP in drug delivery. The development of an amino acid z-scale and the calculation of peptide descriptors was important to understand some factors impacting penetration ability. We believe this method is of great value for pharmaceutical design using CPP for drug delivery. Given the results of this work, we intend to continue studying this approach and these conjugates, and to carry out in vivo experiments, considering Gem-Cys-pVEC our therapeutic lead as it showed the most promising results regarding in silico calculated properties, pharmacokinetic potential and in vitro bioactivity.

## Figures and Tables

**Table 1:** Extracted z-scores for the 20 natural amino acids and their physicochemical properties.

Amino Acid	z1	z2	z3	Amino Acid Properties
Ala (A)	−3.4535	−0.8314	0.8710	Non-polar, aliphatic
Arg (R)	5.9227	−0.7707	1.9428	Positively charged
Asn (N)	0.4104	−4.0436	−0.5900	Polar
Asp (D)	0.1502	−2.2592	−1.7815	Negatively charged
Cys (C)	−1.8132	0.7809	−0.0062	Polar
Gln (Q)	2.5410	−2.5906	0.0520	Polar
Glu (E)	1.4594	−1.6961	−1.7366	Negatively charged
Gly (G)	−3.2706	−1.7938	0.5308	Non-polar, aliphatic
His (H)	1.4195	0.2462	0.6037	Positively charged
Ile (I)	−1.3560	1.8903	0.6660	Non-polar, aliphatic
Leu (L)	−1.5348	1.8836	0.7144	Non-polar, aliphatic
Lys (K)	2.7685	0.6670	2.5607	Positively charged
Met (M)	−0.0676	2.3168	0.4266	Non-polar, aliphatic
Phe (F)	−0.0247	2.9087	−1.2433	Aromatic
Pro (P)	−3.3838	−0.4244	−0.0096	Polar
Ser (S)	−1.6519	−1.4774	0.3484	Polar
Thr (T)	−1.3364	−0.5600	0.3103	Polar
Trp (W)	1.7531	3.3182	−1.7406	Aromatic
Tyr (Y)	2.0819	1.7453	−1.1132	Aromatic
Val (V)	−2.9262	0.7588	0.6271	Non-polar, aliphatic

**Table 2:** Extracted z-scores for the studied peptides.

Peptide	z1 (Size and Shape)	z2 (Polarity)	z3 (Charge)
Pen	2.3233	0.4802	0.6731
Cys-Pen	2.0865	0.5016	0.6364
pVEC	1.0880	0.2586	1.0435
Cys-pVEC	0.9411	0.2895	0.9911

**Table 3:** Electronic and hydrophilic properties of the studied CPP.

	Sequence	#AA^1^	#Arg^1^	#Lys^1^	#His^1^	HR^2^ (%)	Pred.^3^	Exp.^4^	Ref.
Pen	RQIKIWFQNRRMKWKK	16	3	4	0	63	+	+	[38]
Cys-Pen	CRQIKIWFQNRRMKWKK	17	3	4	0	59	+	N.D.	
pVEC	LLIILRRRIRKQAHAHSK	18	4	2	2	44	+	+	[39]
Cys-pVEC	CLLIILRRRIRKQAHAHSK	19	4	2	2	42	+	N.D.	

^1^ # indicates number of total or indicated amino acid residues; ^2^ hydrophilic ratio (calculated at www.bachem.com/service-support/peptide-calculator); ^3^ predicted penetration ability; ^4^ experimentally verified penetration ability. N.D.: not determined.

**Table 4:** Biological activity and half-life of the studied molecules.

Compound	Caco-2 IC50/µM [9]	MKN-28 IC50/µM [9]	HT-29 IC50/µM [9]	t_1/2_ (PBS, 37 °C)/h
Gem	>100	>100	>100	>2 [40]
Cys-Pen	>100	>100	>100	N.D.
Cys-pVEC	>100	>100	>100	N.D.
Gem-Cys-Pen	67.13 ± 2.92	46.99 ± 5.91	47.26 ± 11.3	230 [9]
Gem-Cys-pVEC	>100	20.68 ± 6.81	45.20 ± 1.04	42 [9]

N.D.: not determined.

**Table 5:** Input data used in GastroPlus™ to simulate plasma concentration of GEMZAR^®^.

Parameter	Value	Reference/Data Source
Solubility	5.01 mg/mL at pH 7.92	ADMET Predictor^TM^
pKa	3.54 (DrugBank^a^: 3.6)	ADMET Predictor^TM^
LogP	−1.32 (DrugBank^a^: 1.4)	ADMET Predictor^TM^
Dose	1250 mg	FDA (Ref. ID: 3503046)^b^
Effective permeability, P_eff_	0.59 cm/s × 10^−4^	Caco-2 (Nuno Vale Lab)
Blood/plasma ratio	1.12	ADMET Predictor^TM^
Clearance	168 L/h	[41]
Physiology	Human, fasting conditions	FDA (Ref. ID: 3503046)^b^
Body weight (Kg)	70	FDA (Ref. ID: 3503046)^b^

^a^ From Reference 33; ^b^ From highlights of prescribing information [42].

**Table 6:** Predicted pharmacokinetic properties of studied molecules determined with GastroPlus™.

Compound	F_up_^1^ (%)	B/P ratio^2^	V_c_^3^	F (%) ^4^	Fa (%) ^5^	AUC 0-inf ^6^	AUC 0-t ^7^	C_max_^8^	C_max liver_^9^	Sol. ^10^
Gem	84.61	1.12	1.45	99.949	99.949	7.4368	7.4367	5.9505	5.8709	5.01
Pen	22.50	0.93	0.14	99.999	99.999	7.4404	7.4404	7.4403	7.4398	1.83
Cys-Pen	26.72	0.92	0.11	99.999	99.999	7.4404	7.4404	7.4401	7.4394	4.66
Gem-Cys-Pen	12.91	0.98	0.17	100.000	100.000	7.4405	7.4404	7.4404	7.4403	1.26
pVEC	13.35	1.13	0.16	100.000	100.000	7.4405	7.4405	7.4404	7.4403	28.94
Cys-pVEC	15.53	1.12	0.11	100.000	100.000	7.4405	7.4404	7.4404	7.4403	17.34
Gem-Cys-pVEC	42.89	1.20	0.18	99.998	99.999	7.4404	7.4403	7.4400	7.4393	5.39

^1^ percentage of drug that is not bound to plasma proteins (100 = fully unbound); ^2^ blood/plasma concentration ratio (ratio of concentrations of the drug in whole blood and plasma); ^3^ distribution volume, in L/kg; ^4^ bioavailability; ^5^ fraction absorbed as a percent of the dose (crossing the lumen and entering enterocytes); ^6^ area under the plasma concentration–time curve, in µg-h/mL, extrapolated to infinity; ^7^ area under the plasma concentration–time curve, in µg-h/mL, for the time of the simulation; ^8^ maximum plasma concentration reached in the central compartment, in µg/mL; ^9^ maximum concentration reached in the liver, in µg/mL; ^10^ solubility in mg/mL.

## References

[1] Rybka J., Jurczak W., Giza A., Paszkiewicz-Kozik E., Kumiega B., Drozd-Sokolowska J., Butrym A., Kuliczkowski K., Wrobel T. (2015). Gemcitabine-Based Treatment in Poor-Prognosis Patients with Relapsed and Refractory Hodgkin Lymphoma and Non-Hodgkin Lymphoma--a Multicenter Polish Experience. Adv. Clin. Exp. Med..

[2] Wong A., Soo R.A., Yong W.P., Innocenti F. (2009). Clinical pharmacology and pharmacogenetics of gemcitabine. Drug Metab. Rev..

[3] Mini E., Nobili S., Caciagli B., Landini I., Mazzei T. (2006). Cellular pharmacology of gemcitabine. Ann. Oncol..

[4] Ciccolini J., Mercier C., Dahan L., Andre N. (2011). Integrating pharmacogenetics into gemcitabine dosing--time for a change?. Nat. Rev. Clin. Oncol..

[5] Marechal R., Mackey J.R., Lai R., Demetter P., Peeters M., Polus M., Cass C.E., Young J., Salmon I., Deviere J. (2009). Human equilibrative nucleoside transporter 1 and human concentrative nucleoside transporter 3 predict survival after adjuvant gemcitabine therapy in resected pancreatic adenocarcinoma. Clin. Cancer. Res..

[6] Huang P., Chubb S., Hertel L.W., Grindey G.B., Plunkett W. (1991). Action of 2’,2’-difluorodeoxycytidine on DNA synthesis. Cancer Res..

[7] Alvarellos M.L., Lamba J., Sangkuhl K., Thorn C.F., Wang L., Klein D.J., Altman R.B., Klein T.E. (2014). PharmGKB summary: Gemcitabine pathway. Pharm. Genom..

[8] Kroep J., Van Moorsel C., Veerman G., Voorn D., Schultz R., Worzalla J., Tanzer L., Merriman R., Pinedo H., Peters G. (1998). Role of Deoxycytidine Kinase (dCK), Thymidine Kinase 2 (TK2), and Deoxycytidine Deaminase (dCDA) in the Antitumor Activity of Gemcitabine (dFdC). Purine and Pyrimidine Metabolism in Man Ix.

[9] Vale N., Ferreira A., Fernandes I., Alves C., Araujo M.J., Mateus N., Gomes P. (2017). Gemcitabine anti-proliferative activity significantly enhanced upon conjugation with cell-penetrating peptides. Bioorg. Med. Chem. Lett..

[10] Copolovici D.M., Langel K., Eriste E., Langel U. (2014). Cell-penetrating peptides: Design, synthesis, and applications. ACS Nano.

[11] Nan Y.H., Park I.S., Hahm K.S., Shin S.Y. (2011). Antimicrobial activity, bactericidal mechanism and LPS-neutralizing activity of the cell-penetrating peptide pVEC and its analogs. J. Pept. Sci..

[12] Liu C., Tai L., Zhang W., Wei G., Pan W., Lu W. (2014). Penetratin, a potentially powerful absorption enhancer for noninvasive intraocular drug delivery. Mol Pharm.

[13] Reissmann S. (2014). Cell penetration: Scope and limitations by the application of cell-penetrating peptides. J. Pept. Sci..

[14] Silva S., Almeida A.J., Vale N. (2019). Combination of Cell-Penetrating Peptides with Nanoparticles for Therapeutic Application: A Review. Biomolecules.

[15] Wang F., Wang Y., Zhang X., Zhang W., Guo S., Jin F. (2014). Recent progress of cell-penetrating peptides as new carriers for intracellular cargo delivery. J. Control. Release.

[16] Regberg J., Srimanee A., Langel U. (2012). Applications of cell-penetrating peptides for tumor targeting and future cancer therapies. Pharmaceuticals (Basel).

[17] Stewart K.M., Horton K.L., Kelley S.O. (2008). Cell-penetrating peptides as delivery vehicles for biology and medicine. Org. Biomol. Chem..

[18] Tang H., Yin L., Kim K.H., Cheng J. (2013). Helical Poly(arginine) Mimics with Superior Cell-Penetrating and Molecular Transporting Properties. Chem. Sci..

[19] Wold S. (1976). Pattern recognition by means of disjoint principal components models. Pattern Recognit.

[20] Kirschner G.L., Kowalski B.R. (1978). The application of pattern recognition to drug design. Drug Design.

[21] Hällbrink M., Kilk K., Elmquist A., Lundberg P., Lindgren M., Jiang Y., Pooga M., Soomets U., Langel Ü. (2005). Prediction of cell-penetrating peptides. Int. J. Pept. Res. Ther..

[22] Sandberg M., Eriksson L., Jonsson J., Sjostrom M., Wold S. (1998). New chemical descriptors relevant for the design of biologically active peptides. A multivariate characterization of 87 amino acids. J. Med. Chem..

[23] Hellberg S., Sjostrom M., Skagerberg B., Wold S. (1987). Peptide quantitative structure-activity relationships, a multivariate approach. J. Med. Chem..

[24] Jonsson J., Norberg T., Carlsson L., Gustafsson C., Wold S. (1993). Quantitative sequence-activity models (QSAM)—tools for sequence design. Nucleic Acids Res..

[25] Hansen M., Kilk K., Langel U. (2008). Predicting cell-penetrating peptides. Adv Drug Deliv Rev.

[26] Gautam A., Chaudhary K., Kumar R., Sharma A., Kapoor P., Tyagi A., Raghava G.P., Open Source Drug Discovery Consortium (2013). In silico approaches for designing highly effective cell penetrating peptides. J. Transl. Med..

[27] Lata S., Sharma B.K., Raghava G.P. (2007). Analysis and prediction of antibacterial peptides. BMC Bioinformatics.

[28] Sanders W.S., Johnston C.I., Bridges S.M., Burgess S.C., Willeford K.O. (2011). Prediction of cell penetrating peptides by support vector machines. PLoS Comput. Biol..

[29] Camacho F.L., Torres R., PolláN R.R., Eduardo R., Natasha L., Juan D.G.-A., Jorge B. (2015). Classification of Antimicrobial Peptides with Imbalanced Datasets. Proceedings of 11th International Symposium on Medical Information Processing and Analysis, Cuenca, Ecuador, 17–19 November 2015.

[30] Maccari G., Di Luca M., Nifosi R., Cardarelli F., Signore G., Boccardi C., Bifone A. (2013). Antimicrobial peptides design by evolutionary multiobjective optimization. PLoS Comput. Biol..

[31] Hosea N.A., Jones H.M. (2013). Predicting pharmacokinetic profiles using in silico derived parameters. Mol Pharm.

[32] GEMZAR® (Gemcitabine HCl) for injection; FDA label. https://www.accessdata.fda.gov/drugsatfda_docs/label/2011/020509s069lbl.pdf.

[33] Gemcitabine on DrugBank Database. https://www.drugbank.ca/drugs/DB00441.

[34] Wishart D.S., Feunang Y.D., Guo A.C., Lo E.J., Marcu A., Grant J.R., Sajed T., Johnson D., Li C., Sayeeda Z. (2018). DrugBank 5.0: A major update to the DrugBank database for 2018. Nucleic Acids Res..

[35] Thelen K., Coboeken K., Willmann S., Burghaus R., Dressman J.B., Lippert J. (2011). Evolution of a detailed physiological model to simulate the gastrointestinal transit and absorption process in humans, part 1: Oral solutions. J. Pharm. Sci..

[36] Thelen K., Coboeken K., Willmann S., Dressman J.B., Lippert J. (2012). Evolution of a detailed physiological model to simulate the gastrointestinal transit and absorption process in humans, part II: Extension to describe performance of solid dosage forms. J. Pharm. Sci..

[37] Parrott N.J., Yu L.J., Takano R., Nakamura M., Morcos P.N. (2016). Physiologically Based Absorption Modeling to Explore the Impact of Food and Gastric pH Changes on the Pharmacokinetics of Alectinib. AAPS J..

[38] Derossi D., Calvet S., Trembleau A., Brunissen A., Chassaing G., Prochiantz A. (1996). Cell internalization of the third helix of the Antennapedia homeodomain is receptor-independent. J. Biol. Chem..

[39] Elmquist A., Lindgren M., Bartfai T., Langel U. (2001). VE-cadherin-derived cell-penetrating peptide, pVEC, with carrier functions. Exp. Cell Res..

[40] Tsume Y., Drelich A.J., Smith D.E., Amidon G.L. (2017). Potential Development of Tumor-Targeted Oral Anti-Cancer Prodrugs: Amino Acid and Dipeptide Monoester Prodrugs of Gemcitabine. Molecules.

[41] Peters G.J., Clavel M., Noordhuis P., Geyssen G.J., Laan A.C., Guastalla J., Edzes H.T., Vermorken J.B. (2007). Clinical phase I and pharmacology study of gemcitabine (2′, 2′-difluorodeoxycytidine) administered in a two-weekly schedule. J. Chemother..

[42] https://www.accessdata.fda.gov/drugsatfda_docs/label/2014/020509s077lbl.pdf.

